# Expression, prognosis, immunological infiltration, and DNA methylation of members of the SFRP gene family in colorectal cancer: a comparative bioinformatic and experimental analysis

**DOI:** 10.1007/s11626-024-00998-w

**Published:** 2024-12-27

**Authors:** Haicheng Yang, Zhuo Han, Ying Yang, Shuai Zhou, Bo Zhang, Jiaxing He, Xianli He, Nan Wang

**Affiliations:** Department of General Surgery, Tangdu Hospital, The Air Force Medical University, Xi’an, 710038 China

**Keywords:** SFRP, Immune infiltration, DNA methylation, Colorectal cancer

## Abstract

This study aimed to investigate the expression, prognostic significance, methylation, and immune invasion levels of secreted frizzled-related proteins (SFRP1-5) in colorectal cancer (CRC). Additionally, the relationship between SFRP1/2 methylation and immune infiltration in CRC was explored. The expression of SFRP1-5 was analyzed using several databases, including GEO, TCGA, TIMER, STRING, and GEPIA. Molecular interactions with SFRPs were examined via Cytoscape software. Gene Ontology (GO) and Kyoto Encyclopedia of Genes, and Genomes (KEGG) pathway analyses were conducted using the DAVID database. Methylation levels of SFRP1/2 in CRC were assessed through methylation-specific PCR (MSP) and bisulfite sequencing PCR (BSP) experiments. Apoptosis and proliferation in CRC cells following the knockdown of SFRP1/2 expression were evaluated using flow cytometry and CCK-8 assays. The TISIDB database was used to analyze the relationship between SFRP1/2 methylation levels and immune infiltration. The expression of SFRP1, SFRP2, and SFRP5 was significantly lower in CRC patients, while SFRP4 expression was higher compared to that in healthy individuals. Elevated mRNA expression of SFRP2 was significantly associated with improved overall survival (OS), disease-specific survival, and progression-free intervals. SFRP1/2 expression was also linked to immune invasion, with higher levels correlating with increased immune infiltration. Both SFRP1 and SFRP2 showed hypermethylation in CRC. Knockdown of SFRP1/2 expression resulted in increased proliferation of CRC cells, and their methylation levels were inversely correlated with immune cell presence. The expression, methylation, and immune cell infiltration patterns of the SFRP family in CRC differed markedly from those in healthy individuals. These findings suggest that SFRPs may serve as potential therapeutic targets and key genes associated with immune cell infiltration in CRC.

## Introduction

Colorectal cancer (CRC) is a prevalent malignancy of the gastrointestinal tract, responsible for approximately 600,000 deaths annually worldwide. The incidence is higher in Western countries than in Asian and African nations (Baidoun *et al*. 2020; Patel *et al*. 2022). Moreover, CRC incidence has been steadily increasing (Messersmith 2019). Many patients are diagnosed at advanced stages due to nonspecific early symptoms, which contribute to a poor prognosis and reduced treatment efficacy, with a 5-yr overall survival (OS) rate below 4% (Li *et al*. 2021b). Diagnostic methods include computed tomography imaging, nuclear medicine imaging, endoscopic evaluation, and biopsy (Piawah and Venook 2019). Despite these diagnostic techniques, there remains a need to identify novel biomarkers as diagnostic indicators to guide personalized treatment and improve patients’ OS and disease-free survival (DFS).

Secreted frizzled-related proteins (SFRPs) are extracellular tumor suppressor genes involved in the Wnt signaling pathways, playing crucial roles in embryogenesis and tumorigenesis. SFRPs are modular proteins composed of secreted signal peptides and cysteine-rich domains (Hsueh *et al*. 2021). These proteins bind directly to Wnt ligands or frizzled receptors, thereby inhibiting the initiation of signal cascades. Numerous studies have shown that abnormal SFRP expression is linked to the development and progression of various malignancies (Cheng *et al*. 2017b). Furthermore, SFRP methylation has been implicated in several cancers, including cervical cancer (Lin *et al*. 2009), hepatocellular carcinoma (Takagi *et al*. 2008), and renal cancer (Kawakami *et al*. 2011). However, the expression, mechanisms, and prognostic implications of SFRP family members in CRC remain poorly understood.

This study performed bioinformatics analyses on publicly available CRC databases and integrated these with experimental data obtained in this project. We analyzed and reported the differential expression, prognostic significance, functional enrichment, immune cell infiltration, and methylation levels of different SFRP family members in CRC. Additionally, we examined the effects of SFRPs on CRC cell proliferation and apoptosis to deepen the understanding of the mechanisms underlying the involvement of SFRP gene family members in CRC.

## Materials and methods

### SFRPs gene family expression in the GEPIA, TCGA, and GEO

The GEPIA (Gene Expression Profiling Interactive Analysis, http://gepia.cancer-pku.cn.) database was used to investigate the expression of SFRPs in CRC, drawing on data from TCGA and the Genotype-Tissue Expression (GTEx, https://www.genome.gov/Funded-Programs-Projects/Genotype-Tissue-Expression-Project.). Expression levels of each SFRP family member were analyzed in cancer and normal tissues within GEPIA. Additionally, mRNA expression data from normal colorectal tissues and CRC tissues were retrieved from the GEO database (https://www.ncbi.nlm.nih.gov/geo), using datasets GSE23878, GSE113513, GSE79793, and GSE156355. The *limma* package in R language was used to analyze the mRNA expression levels of SFRP1, SFRP2, FRZB, SFRP4, and SFRP5, producing a differential expression heatmap.

### Survival analysis

RNAseq data from the TCGA-COAD and TCGA-READ STAR pipelines were downloaded and collated from the TCGA database (https://portal.gdc.cancer.gov), with data extracted in TPM format, along with associated clinical information. Using R, the *survival* package was applied to perform proportional hazards assumption testing and fit a survival regression model. Results were visualized with the *Survminer* and *ggplot2* packages. For optimal grouping, the cutpoint function in the *Survminer* package was used to identify the best cutoff point for group stratification.

### Analyze the molecular interactions through STRING and KEGG/gene ontology (GO) analysis

The molecular interactions and correlations between SFRPs and related proteins were analyzed using the STRING (https://string-db.org/) online interactive tool. It should be noted that the input genes used to identify the internal communications were as follows: ARMC8; RANBP9; GID8; SFRP2; RMND5A; MAEA; RMND5B; DVL2; FZD1; FZD7; FZD6; WNT1; LRP5; FZD4; FZD2; ROR2; WNT3A; WNT5A; FZD5; LRP6; FRZB; WNT2; WNT7A; SFRP1; RYK; NDP; FZD8; FZD9; MET; ROR1; TNFSF11; SFRP4; SFRP5; TNFRSF11A; and TNFRSF11B. The network contained 35 nodes and 470 edges, with a local clustering coefficient of 0.883 and a PPI (protein–protein interaction) enrichment *p*-value of less than 1.0e-16.

For output data analysis, Cytoscape software was used for further experimental examination. The candidate genes were analyzed using the DAVID online tool (https://david.ncifcrf.gov/) for GO and KEGG pathway analyses. GO analysis categorized the candidate genes by molecular function, cellular location, and biological pathways. The “ggplot2” package was employed to create a bubble chart.

### Analyze the immune infiltration through the TIMER and TISIDB analysis

The relationship between the expression of various molecules in tumors and immune-related markers was evaluated using TIMER (Tumor IMmune Estimation Resource, https://cistrome.shinyapps.io/timer), based on data from The Cancer Genome Atlas (TCGA). TIMER was used to analyze the infiltration of SFRP family members in relation to CD4 + T cells, CD8 + T cells, macrophages, neurons, and B cells. Additionally, we used the TISIDB platform (http://cis.hku.hk/TISIDB/index.php) to analyze gene expression and examine the relationship between methylation levels and tumor-infiltrating lymphocytes, specifically focusing on the methylation status of SFRP1 and SFRP2 and their association with immune infiltration.

### Methylation data and processing

DNA methylation data were derived from RNAseq and Illumina human methylation data in the TCGA database. Using NCBI, we identified the 2000-bp upstream promoter region and the 200-bp downstream region of the SFRP1 and SFRP2 genes. MethPrimer (http://www.urogene.org/cgi-bin/methprimer/methprimer.cgi) was then used to predict CpG islands within the promoter regions of these genes. Additionally, we analyzed the TCGA database to explore the relationship between SFRP1/2 expression and the expression of DNA methyltransferases (DNMTs), including DNMT1, DNMT3A, and DNMT3B.

### Cell culture

CRC cell lines (SW620, HCT116, LoVo, and COLO205), as well as the normal colon cell line NCM460, were obtained from Procell in Wuhan, China. These cell lines were cultured in DMEM medium supplemented with 1% penicillin–streptomycin and 10% fetal bovine serum (both from Gibco, Thermo Fisher Scientific, Grand Island, NY). Standard cell culture protocols were followed for cryopreservation, resuscitation, passage, culture, and identification. Plasmid transfection was conducted using Lipofectamine 3000 as the transfection reagent.

### RNA extraction and qRT-PCR

The processed cells were lysed in 1 ml of Trizol for 5 minutes, followed by the addition of 200 ml of chloroform. The cells were inverted approximately 30 times, and then centrifuged at 12,000 rpm for 5 min at 4°C. The upper aqueous layer was carefully transferred to an enzyme-free EP tube, and an equal volume of isopropanol was added. After centrifuging for 14 min at 12,000 rpm, the upper liquid was removed, leaving the white RNA pellet at the bottom.

RQ1 RNase-Free DNase (Promega, Madison, WI) was used to treat the RNA. The reaction mixture included 10 μl of solution containing 1 μl of 10 × buffer, 2 μl of DNase, 2 μg of RNA, and diethylpyrocarbonate (DEPC) water. The solution was incubated at 37°C for 30 min, and then immediately placed on ice. Next, 1 μl of RQ1 DNase Stop Solution was added to each tube, followed by incubation at 65°C for 10 min. The RNA was then reverse-transcribed into cDNA, which was incubated at 37°C for 2 min, 42°C for 60 min, and 70°C for 5 min, before being stored at 4°C.

The obtained cDNA was subjected to quantitative reverse transcription PCR (qRT-PCR). The PCR primer sequences are depicted in Table 1. The qRT-PCR was performed under the following conditions: initial denaturation at 95°C for 10 min, followed by 40 cycles of denaturation at 95°C for 15 s and annealing/extension at 60°C for 30 s. The details of the reaction system are provided in Table 2. The Ct values were then calculated to determine the relative mRNA expression levels.

**Table 1.** Primer sequence

Primers	Sequence (5′−3′)
SFRP1	F: CCCACCTTTCAGTCCGTGTT
	R: AATGCTGCAAGAACAAGCCG
SFRP2	F: GAAGTTCCTGTGCTCGCTCT
	R: GCCACAGCACCGATTTCTTC
GAPDH	F: CAGCCTCAAGATCATCAGCA
	R: TGTGGTCATGAGTCCTTCCA

*F*, forward; *R*, reverse

**Table 2.** qRT-PCR system

Reaction system	Volume
2*Quantinova SYBR Green mix	10 µl
Primer A (reverse)	1 µl
Primer B (forward)	1 µl
Rnase-free water	6.5 µl
cDNA	1.5 µl
Total volume	20 µl

### Demethylation of CRC cells and colon cells by 5-azaD (5-aza-2-deoxycytidine)

5-azaD (Sigma-Aldrich, St. Louis, MO) was dissolved in water and then diluted with the cell culture medium. The cell lines were treated with 5-azaD for 4 d, with the medium being replaced daily. After treatment, the demethylated cells were directly collected for total RNA isolation, CCK-8 assays, and apoptosis analysis. Cells cultured under normal conditions and treated with 0.2% DMSO served as the control group.

### MSP (methylation-specific PCR) assay

Genomic DNA was extracted from cells and colon tissues using the Genome DNA Purification Kit (Tiangen, Beijing, China). The DNA was then modified according to the instructions provided with the EZ DNA Methylation Kit (ZYMO RESEARCH, Orange, CA). Primers for MSP were designed as follows:

- Methylation-specific primers for SFRP1:
  - Forward: 5′-TAGTAAATCGATCGTGC-3′
  - Reverse: 5′-AACTCCTACGACGAACC-3′

- Methylation-specific primers for SFRP2:
  - Forward: 5′-TTTGTTTATTGGGGGGGC-3′
  - Reverse: 5′-CCTCACGCTTACTAAAAAA-3′

- Non-methylation-specific primers for SFRP1:
  - Forward: 5′-TTTAGTAAATTGAATTTTTGT-3′
  - Reverse: 5′-CATTTTTTTTTAAACTCCTACAACCAAAACCC-3′

- Non-methylation-specific primers for SFRP2:
  - Forward: 5′-TTGTTTTTTTGTGGGGGT-3′
  - Reverse: 5′-CCCTCACCACACTTACTAAAAAAA-3′

The PCR conditions were as follows: initial denaturation at 95°C for 5 min, followed by 30 cycles of denaturation at 95°C for 20 s, annealing at 60°C for 30 s, extension at 72°C for 20 s, and a final extension at 75°C for 5 min. The PCR products were then resolved on a 2% agarose gel, stained with ethidium bromide, and visualized under ultraviolet light.

### BSP (bisulfite sequencing PCR) Assay

DNA was extracted from normal colon cells and CRC cells using the DNA Extraction Kit (Axigen, Los Angeles, CA). The extracted DNA was then treated with hydrogen sulfate. PCR products were excised from the gel and ligated into the pMD® 18-T Vector (D101B; Takara, Kusatsu, Japan). The ligated plasmid was transformed into DH5α bacteria, which were cultured overnight. Plasmid DNA was then isolated, and at least ten individual clones were selected for sequence analysis.

### Detection of proliferation in CRC cells by CCK8 assay

SW620 and HCT116 cells were seeded into a 24-well plate at a density of 5 × 10^5^ cells/ml, with a blank control group included. After 12 h of incubation, the control group was removed, and the remaining cells were exposed to plasmids for 48 h (SW620) and 72 h (HCT116). Following treatment, 25 µl of CCK-8 reagent was added to each well. The wells were protected from light and incubated at 37°C for 1.5 h. Absorbance at 450 nm was measured using a microplate reader, and the cell growth rate was calculated using the following formula:

1 $$\text{Growth Rate }\left({\%}\right)=\frac{({\text{A}}_{\text{experimental}}-{\text{A}}_{\text{blank}})}{({\text{A}}_{\text{control}}-{\text{A}}_{\text{blank}})}\times 100\%$$

where A represents the absorbance values obtained from the measurements.

### Detection of apoptosis in CRC cells by flow cytometry

SW620 and HCT116 cells were seeded at a density of 5 × 10^5^ cells/ml in a 6-well plate. After incubation, the cells were washed with pre-cooled PBS, and trypsin was added for digestion. The cells were collected, centrifuged, and resuspended in PBS. To assess cell viability, PI and Annexin V-FITC antibodies were added to the samples. Flow cytometry analysis was then performed in a dark environment.

### Statistical analysis

Statistical analysis was performed using SPSS 21.0, and the data are presented as mean ± standard deviation. Differences among the groups were compared using one-way ANOVA, and pairwise comparisons between groups were evaluated using the *t*-test. A *p*-value of less than 0.05 was considered statistically significant. All experiments were conducted independently at least three times.

## Results

### Differential transcriptional expression of SFRPs in patients with CRC

**Figure 1.**
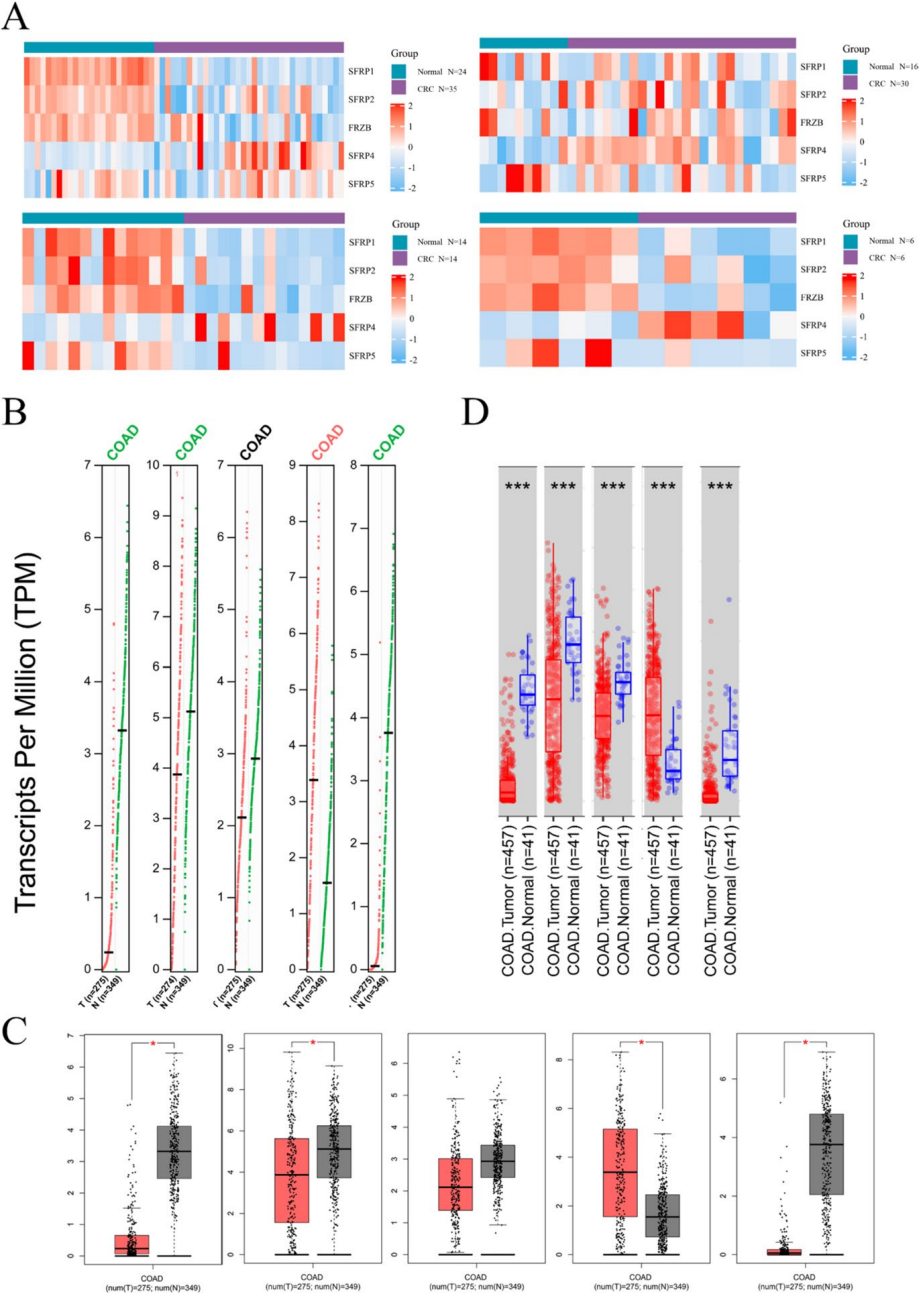
The expression values of SFRP1-5 in colorectal cancer patients compared to normal individuals. (*A*) The differences of SFRP1-5 expression between normal colon tissue and colon cancer tissue in four GEO databases including GSE23878, GSE113513, GSE79793, GSE156355; (*B*) The differential expression of SFRP1-5 in normal colon tissues and colon cancer tissues in GEPIA database; (*C*) The differential expression of SFRP1-5 in TCGA database; and (*D*) The differential expression of SFRP1-5 between normal colon tissue and colon cancer tissue obtained from TIMER database based on TCGA database (*p < 0.05, **p < 0.01, ***p < 0.001).

Our analysis revealed differential expression of SFRPs in CRC compared to normal colorectal tissue. Specifically, SFRP4 showed higher expression levels in CRC, while the other four members of the SFRP family exhibited lower expression levels (Table 3, Fig. 1*A*). Additionally, using the GEPIA and TIMER databases, we observed that SFRP1, SFRP2, and SFRP5 were downregulated in CRC patients compared to normal colorectal tissues, whereas SFRP4 was upregulated. The expression of SFRP3 (FRZB) displayed inconsistency between the two databases, with TIMER showing low expression in CRC patients, while GEPIA and its corresponding TCGA database results showed no significant differences between CRC and normal tissues (Fig. 1*B*, *C*, and *D*).

**Table 3.** Differential expression of SFRPs in colorectal cancer (class: CRC vs. normal)

Expression	Fold change	*p*-value	*t*-statistics	Source
SFRP1	− 3.541	6.47E − 14	− 11.2773	GSE23878
	− 3.641	3.68E − 6	− 6.832	GSE113513
	− 7.911	0.625	− 0.972	GSE79793
	− 2.726	0.001	− 7.317	GSE156355
SFRP2	− 3.241	6.12E − 5	− 4.974	GSE23878
	− 2.446	0.001	− 4.214	GSE113513
	− 9.174	0.618	− 1.015	GSE79793
	− 3.048	0.049	− 3.071	GSE156355
FRZB	− 6.794	4.62E − 5	− 5.062	GSE23878
	− 1.859	4.47E − 5	− 5.667	GSE113513
	− 9.089	0.779	− 0.545	GSE79793
	− 1.165	0.004	− 5.484	GSE156355
SFRP4	1.108	0.009	3.189	GSE23878
	0.869	0.083	2.212	GSE113513
	2.486	0.001	3.631	GSE79793
	2.924	0.117	2.378	GSE156355
SFRP5	− 2.338	0.698	− 0.619	GSE23878
	− 4.14	0.023	− 2.870	GSE113513
	− 7.721	0.587	− 1.483	GSE79793
	− 2.204	0.297	− 1.600	GSE156355

### Analyzing the association between patients’ survival and expression of SFRPs in CRC

**Figure 2.**
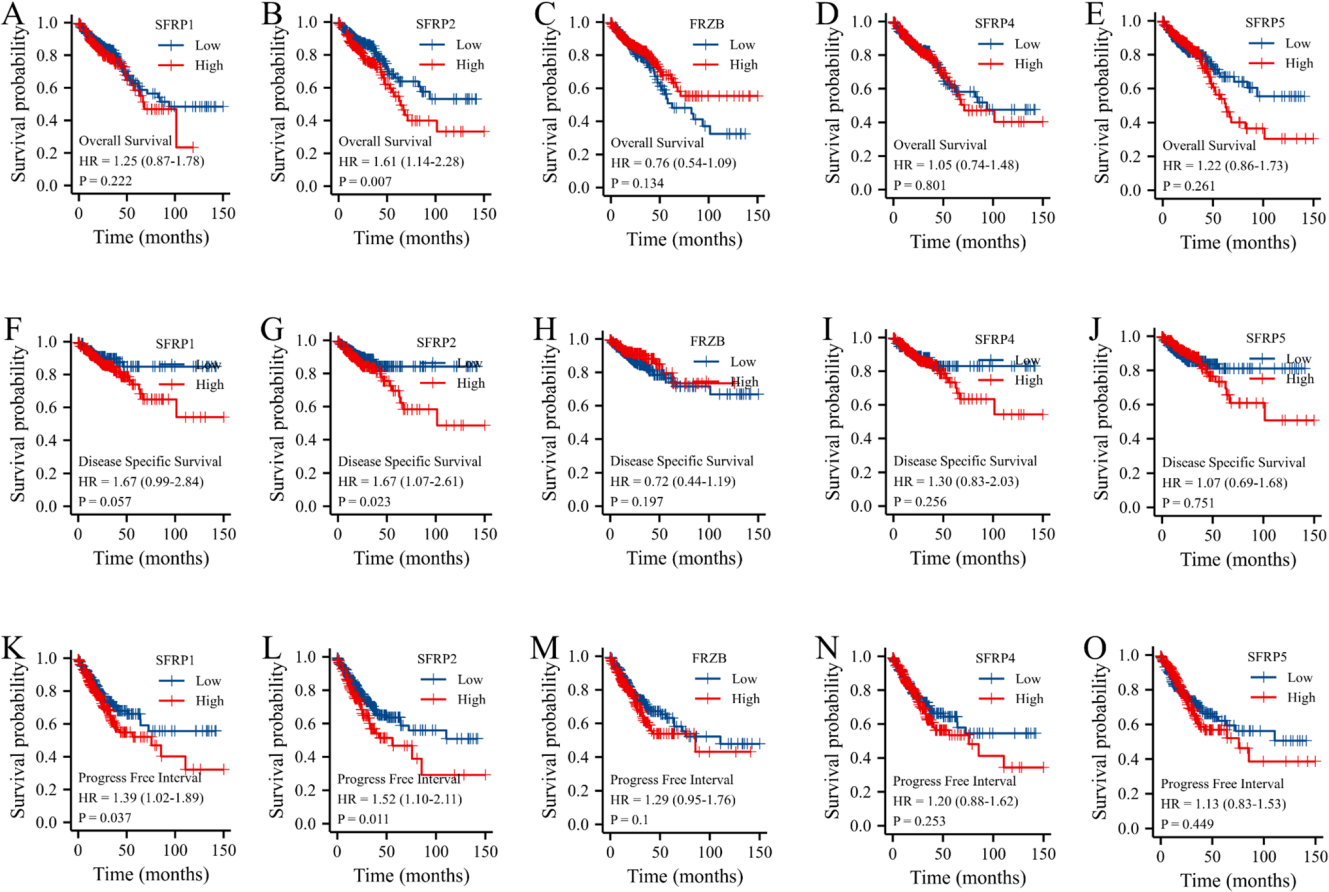
The relation between the SFRP1-5 differential expression with overall survival, disease special survival, and progress-free interval of CRC patients extracted from TCGA database; (*A*–*E*) SFRP1-5 expressions and overall survival; (*F*–*J*) SFRP1-5 expressions and special survival; and (*K*–*O*) SFRP1-5 expressions and progress-free interval.

Survival analysis was conducted using the GEPIA database to further investigate the prognostic significance of the SFRP family in CRC patients. The relationship between the differential expression of SFRP1-5 and OS, disease-specific survival, and progression-free interval in CRC patients, as extracted from the TCGA database, is illustrated in Fig. 2. The findings indicated that increased mRNA expression of SFRP1 was significantly associated with progression-free interval (Fig. 2*K*). Additionally, elevated mRNA expression of SFRP2 was significantly linked to OS, disease-specific survival, and progression-free interval (Fig 2*B*, *G* and *L*). However, the other members of the SFRP family did not show significant associations with OS, disease-specific survival, or progression-free interval.

### Protein interaction network

**Figure 3.**
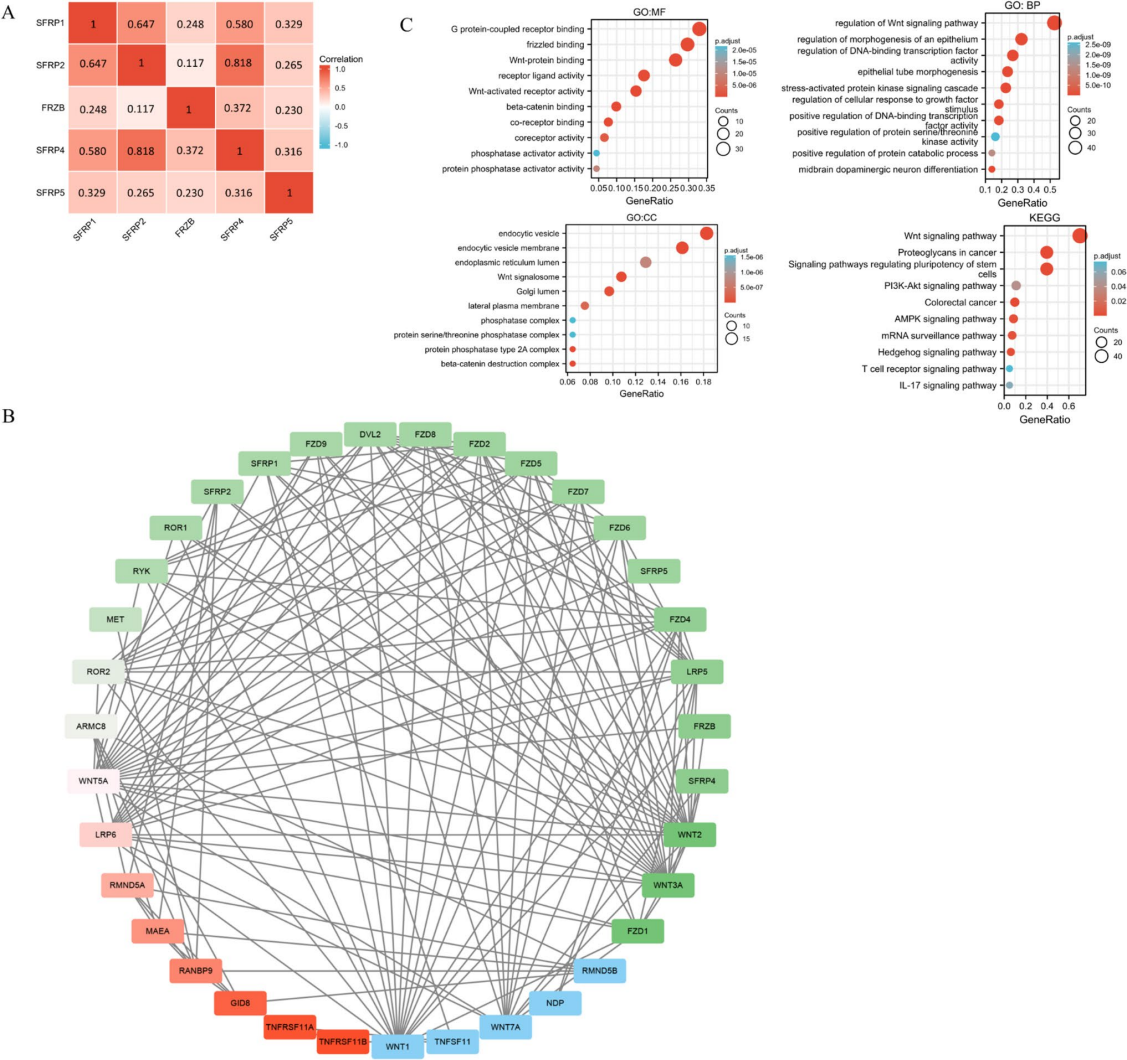
Interaction between SFRPs families and GO/KEGG analysis. (*A*) The co-expression result of SFRP1-5 in colorectal cancer patients; (*B*) the molecular network of SFRP1-5 interactions predicted by STRING database and Cytoscape; and (*C*) the GO and KEGG analysis of SFRP1-5 molecular interaction network. The colors in *panel* *B* represent the degree of enrichment of other genes, with *blue*, *green*, and *red* colors showing high, moderate, and low degrees, respectively.

**Figure 4.**
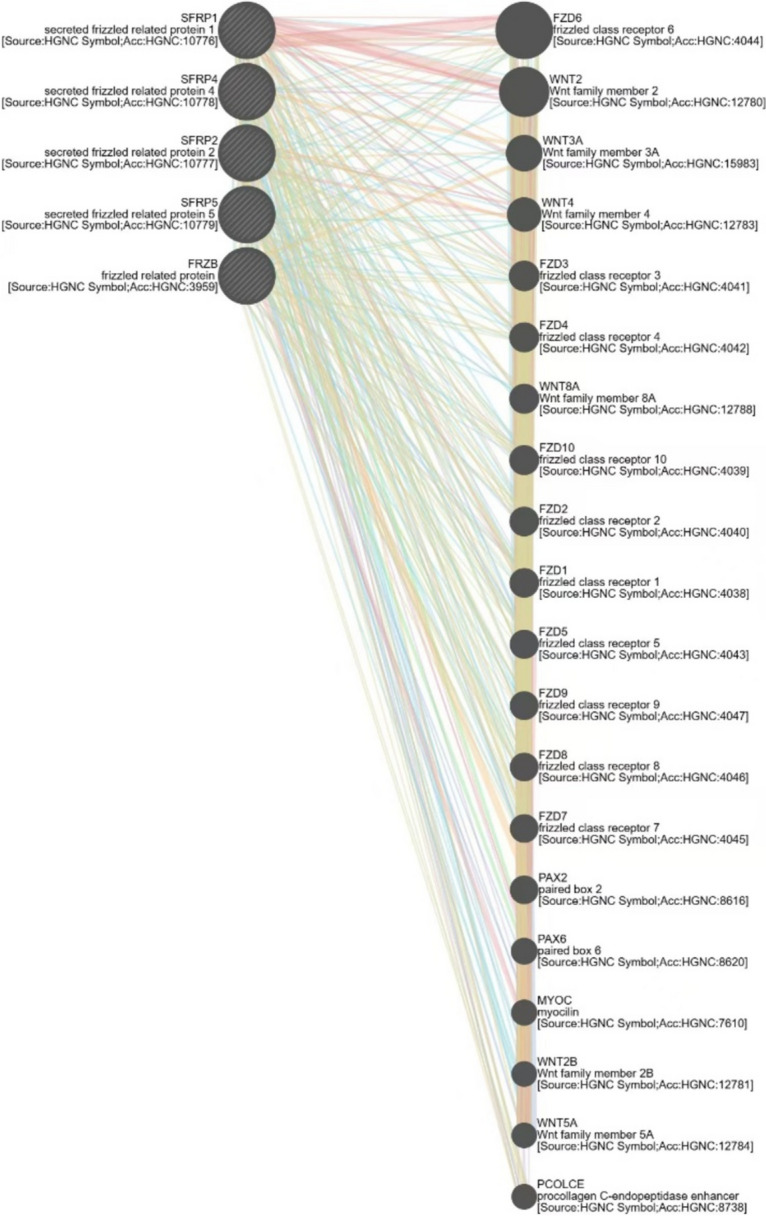
The correlation analysis of GeneMANIA database.

The STRING database was utilized to analyze the molecules associated with SFRPs and their potential interactions to better understand their functional roles. The co-expression of SFRP1-5 in CRC and the PPI network highlighted the potential interactions and connections between the SFRP family and other molecules (Fig. 3*A*, *B*). Notably, all the molecules involved are closely related to the Wnt signaling pathway. Further analysis using the GeneMANIA database revealed that the SFRP family and its associated molecules are closely linked to the Wnt signaling pathway, immune system regulation, and stem cell differentiation. These findings suggest promising avenues for future research. Figure 4 presents the correlation analysis from the GeneMANIA database.

Additionally, analysis using the DAVID database provided insights into the molecular functions, biological processes, and cellular components associated with the SFRP family. These include their involvement in Wnt signal receptor activation, G protein binding, regulation of the Wnt signaling pathway, epithelial morphogenesis, DNA-binding transcription factors, and cellular locations such as endocytic vesicles, endoplasmic reticulum cavities, Golgi bodies, and lateral plasma membranes. KEGG analysis further indicated the involvement of SFRPs in various pathways, including the Wnt signaling pathway, proteoglycans in cancer, signaling pathways regulating stem cell pluripotency, PI3K-Akt signaling pathway, CRC, AMPK signaling pathway, mRNA surveillance pathway, and other pathways related to CRC (Fig. 3*C*).

### Immune infiltration analysis of SFRPs

**Figure 5.**
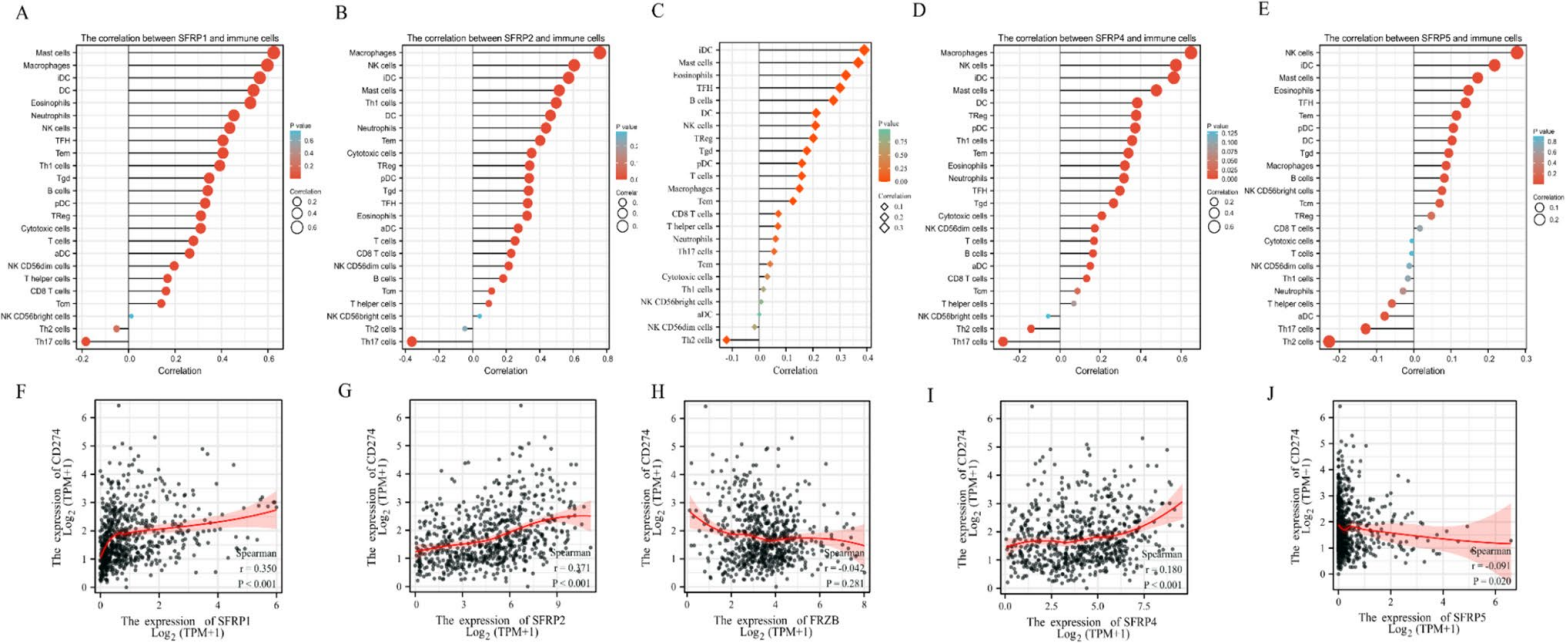
The correlation and expression levels of SFRP family and immune cells. *Panels* *A*–*E* show the correlation between SFRP1-5 with immune molecules and *panels* *F*–*J* show the correlation between SFRPP1-5 with CD274 (PD-L1) expression.

The ssGSEA method was employed to calculate the correlation between the SFRP family and immune cells using the TCGA database for CRC. The correlation and expression levels of the SFRP family and immune cells are presented in Fig. 5. The results show that SFRP1 is primarily associated with mast cells, macrophages, iDC, DC, and eosinophils. SFRP2 is mainly related to macrophages, NK cells, iDC, mast cells, Th1 cells, DC, and neutrophils. SFRP3 is primarily associated with iDC, mast cells, eosinophils, TFH cells, and B cells. Furthermore, SFRP4 is mainly related to macrophages, NK cells, mast cells, DC, and Treg cells. Finally, the findings show that SFRP5 is primarily associated with NK cells, iDC, mast cells, eosinophils, and TFH cells (Fig. 5*A*–*E*).

**Figure 6.**
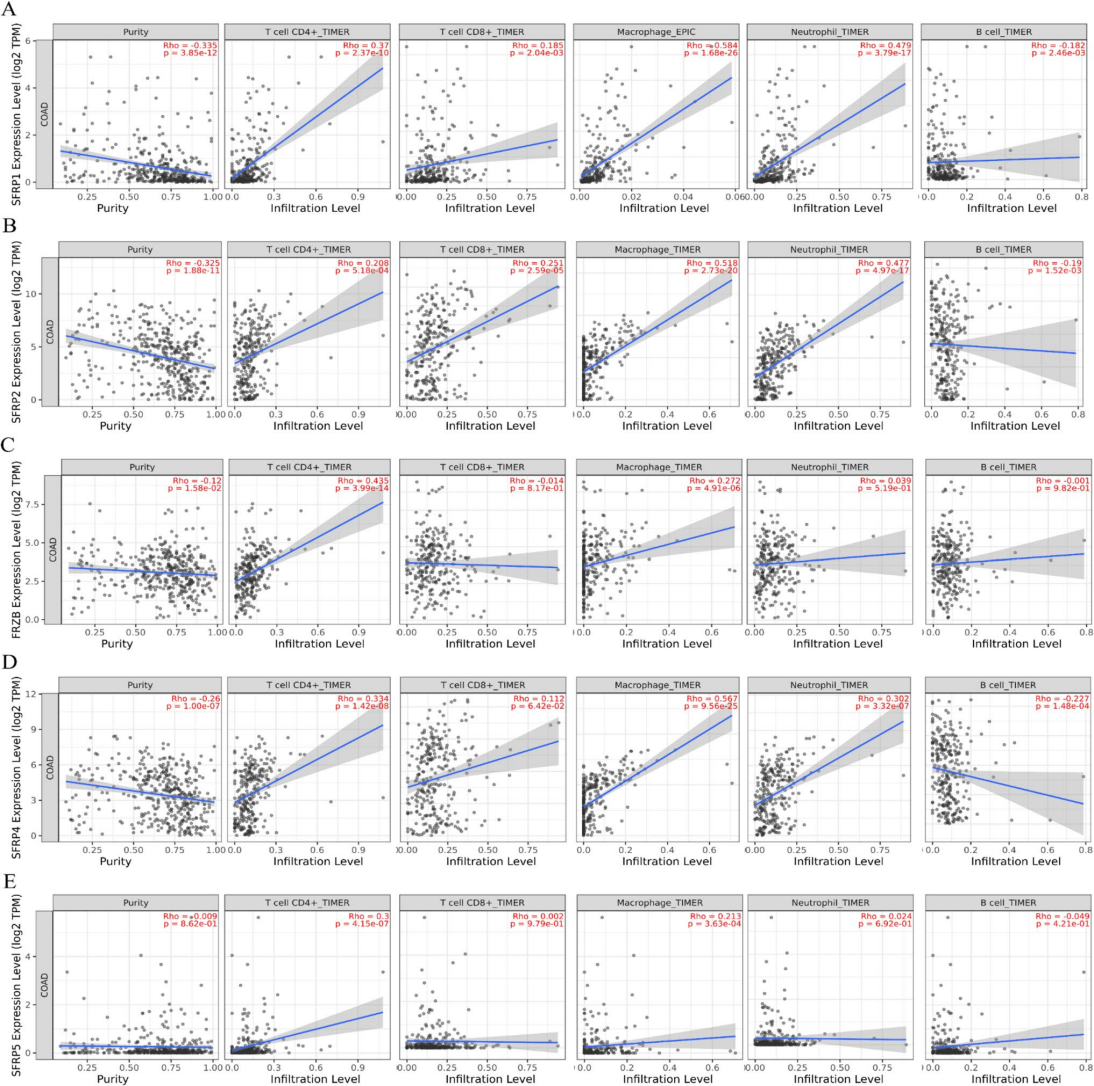
The correlations between SFRP family members with CD4 + T, CD8 + T, macrophages, and neutrophil cells extracted from TIMER database. *Panels* *A*–*E* are the corresponding correlation diagram of SFRP1-5, respectively.

Furthermore, the relationship between the SFRP family and the expression of CD274 (PD-L1) was analyzed. The results showed that SFRP1, SFRP2, and SFRP4 exhibited positive correlations with CD274 expression, while SFRP5 showed a negative correlation (Fig. 4*F*, *G*, *H*, *I*, *J*). Figure 6 presents the correlations between SFRP family members and CD4 + T cells, CD8 + T cells, macrophages, and neutrophils, as extracted from the TIMER database. The results obtained from the TIMER database revealed positive correlations between SFRP1/2/3/4/5 and CD4 + T cells (*p* < 0.01), SFRP1/2 and CD8 + T cells (*p* < 0.01), SFRP1/2/3/4/5 and macrophages (*p* < 0.01), and SFRP1/2/5 and neutrophils (*p* < 0.01). Moreover, SFRP1/2/4 exhibited positive correlations with B cells (*p* < 0.01).

### Methylation level of SFRP1 and SFRP2 and their effects on proliferation and apoptosis of CRC cells

**Figure 7.**
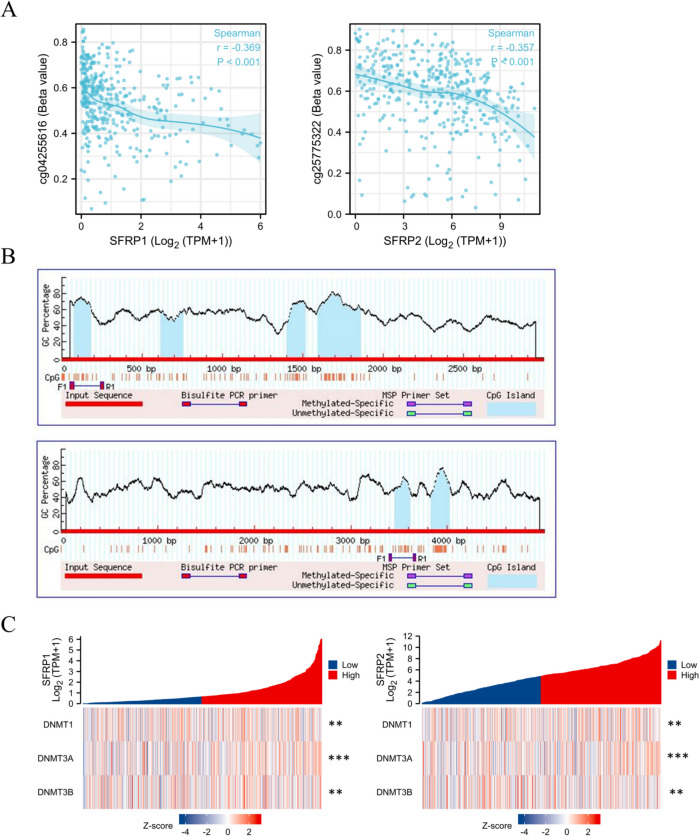
*Panel* *A* shows the correlation between the DNA expression of SFRP1/2 and the methylation level of SFRP1/2, *panel* *B* shows the prediction of the CpG island of SFRP1/2, and *panel* *C* shows the DNA expression of SFRP1/2 and the expression of the methyltransferases DNMT1, DNMT3A, and DNMT3B (**p < 0.01, ***p < 0.001).

Figure 7 shows the correlation between the DNA expression of SFRP1/2 and the methylation levels of SFRP1/2, the prediction of the CpG islands in SFRP1/2, and the expression of the methyltransferases DNMT1, DNMT3A, and DNMT3B, all obtained from the CRC analysis in the TCGA database. The findings revealed a negative correlation between the cg04255616 methylation probe and SFRP1, as well as a negative correlation between the cg25775322 methylation probe and SFRP2 (Fig. 7*A*). Further analysis using the MethPrimer database identified four CpG islands in SFRP1 and two CpG islands in SFRP2 (Fig. 7*B*). Additionally, the TCGA database showed a negative correlation between the expression of SFRP1/2 in CRC and the expression of the methyltransferases DNMT1, DNMT3A, and DNMT3B (Fig. 7*C*).

**Figure 8.**
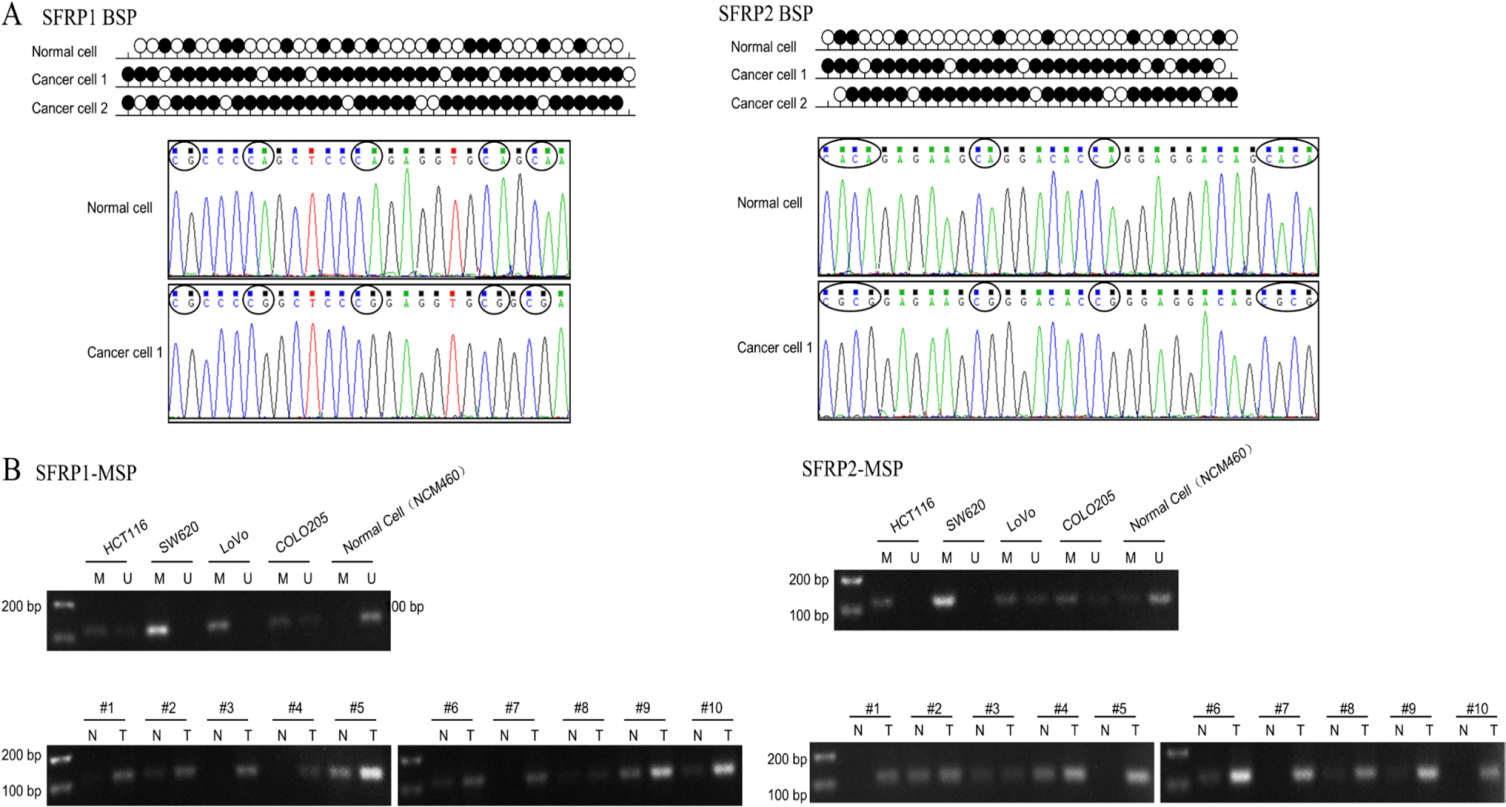
The DNA methylation analysis of SFRP1 promoter region based on BSP sequencing and MSP sequencing. (*A*) DNA methylation analysis of SFRP1 promoter region based on BSP sequencing includes − 351 to − 93 positions and 42 CG sites. The DNA methylation of the samples isolated from colon cancer cells was positive, and the methylation status of normal colon cells was determined as a negative control. (*B*) DNA methylation analysis of SFRP1 promoter region based on MSP sequencing. Detecting the strength/presence of M-bands in 10 paired tumor-normal tissues.

Bisulfite sequencing PCR (BSP) was conducted to determine the methylation levels of SFRP1/2. The analysis revealed significantly increased methylation in the promoter region of SFRP1 in CRC tissues compared to normal colon cells. MSP further confirmed the elevated methylation levels in CRC tissues, along with a corresponding decrease in unmethylated levels in CRC cell lines. Moreover, the expression of SFRP1 and SFRP2 in CRC cells was lower compared to the normal colon cell line NCM460. Figure 8 illustrates the DNA methylation analysis of the SFRP1 promoter region based on BSP and MSP sequencing.

**Figure 9.**
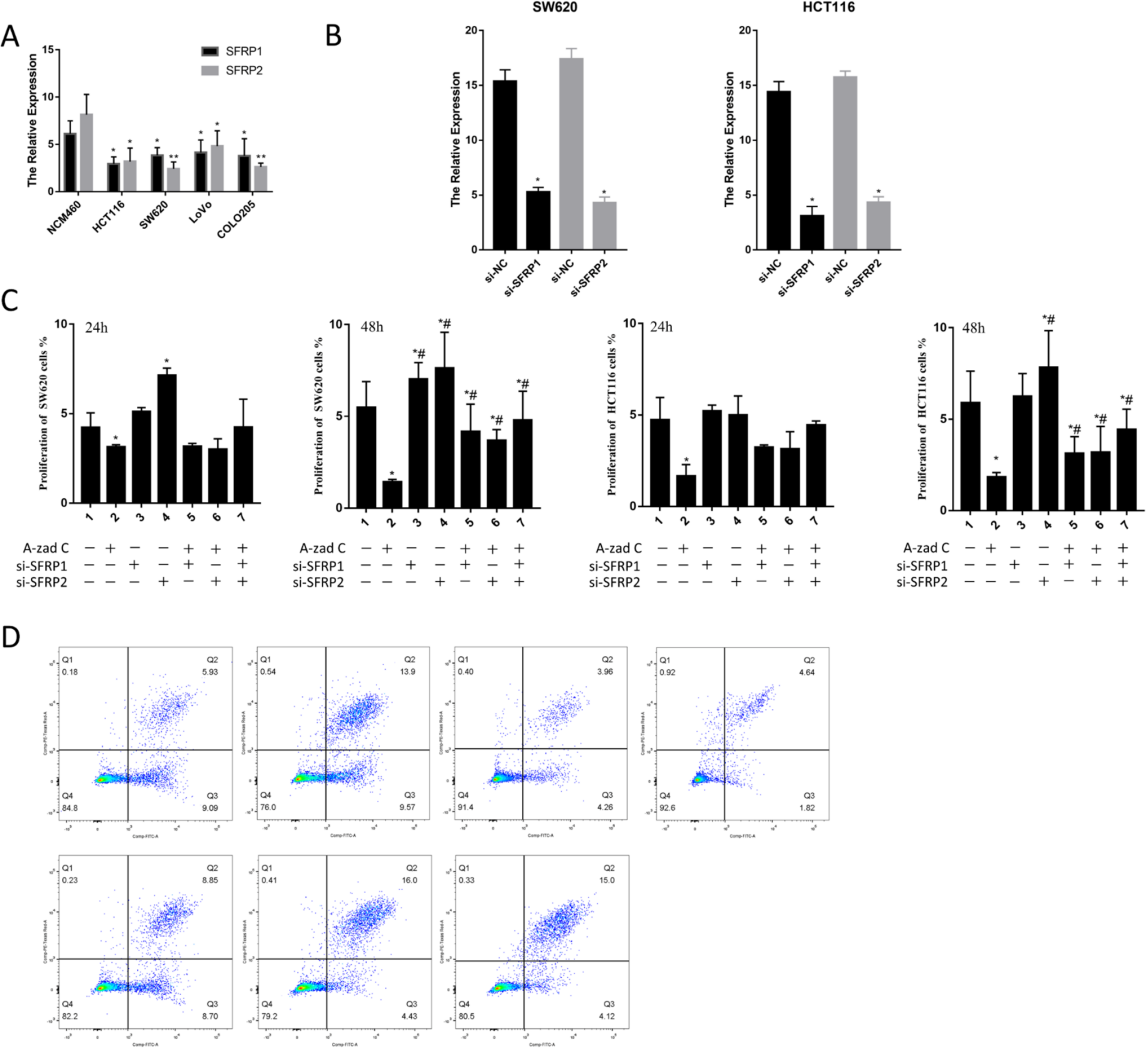
The expression levels of SFRP1/2, proliferation, and apoptosis in different cell lines. (*A*) The expression of SFRP1/2; (*B*) The SFRP1/2 expression after gene knockout in two cell lines, * represents the group compared with the si-NC; (*C*) The cell proliferation after using 5-azaD, si-SFRP1, and si-SFRP2; (*D*) The apoptosis of cells after using 5-azaD, si-SFRP1 and si-SFRP2, * represents the group compared with group 1 and ^#^ represents the group compared with group 2 (*p < 0.05, **p < 0.01, ***p < 0.001).

Figure 9 presents the expression levels of SFRP1/2, along with the effects on proliferation and apoptosis in different cell lines. Subsequent experiments showed that 5-azaD treatment inhibited the proliferation and promoted apoptosis of CRC cells. In contrast, si-SFRP1 and si-SFRP2 treatments promoted cell proliferation and inhibited apoptosis. Notably, the combination of 5-azaD with si-SFRP1/si-SFRP2 partially reduced proliferation and increased apoptosis in CRC cells compared to si-SFRP1/si-SFRP2 treatment alone. While 5-azaD has the potential to influence a wide range of gene expressions, it is important to recognize that the observed results may also reflect the contributions of other factors, in addition to the effects of 5-azaD.

### Relationship between expression of SFRP1, SFRP2, methylation level, and immune infiltration

**Figure 10.**
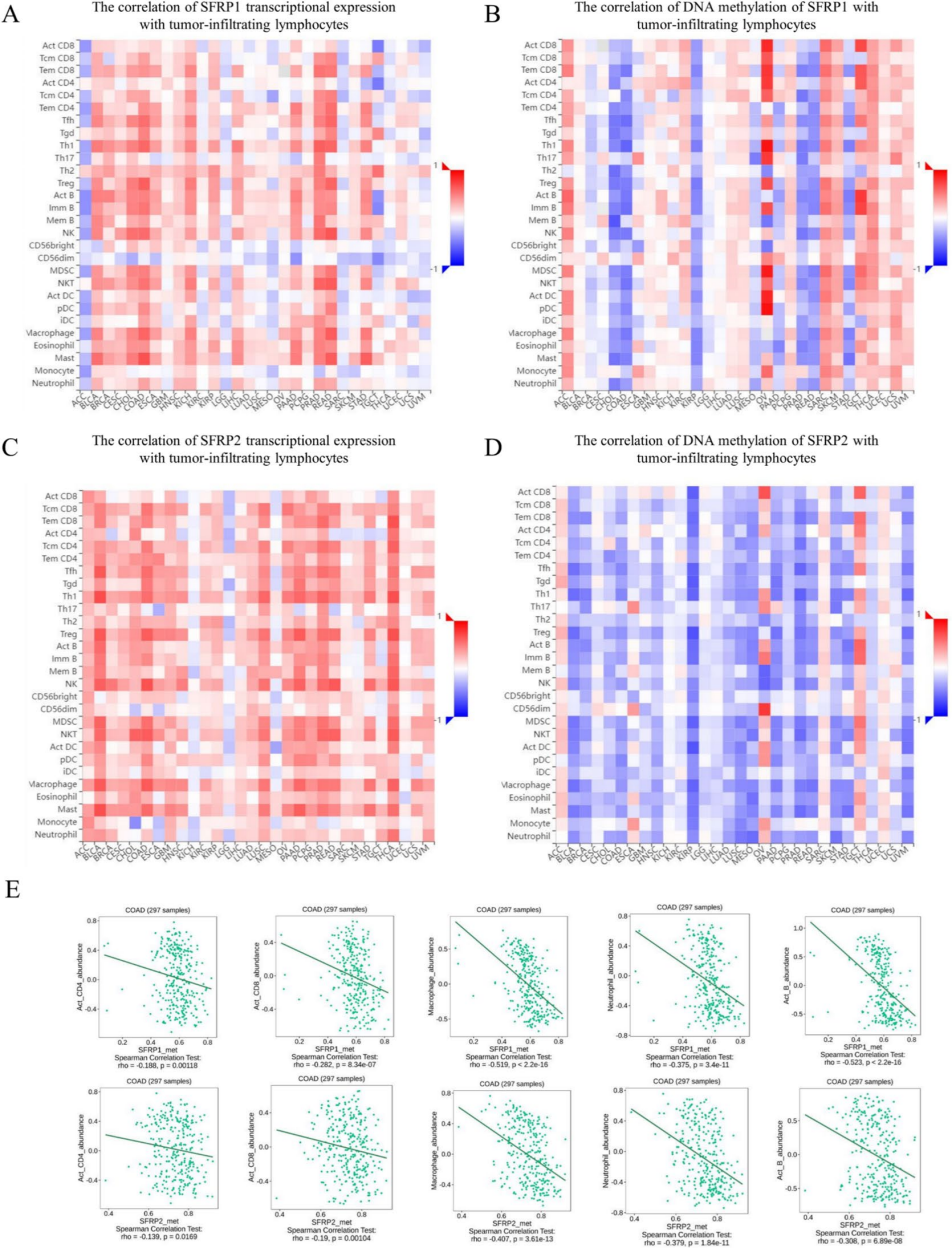
Correlations between SFRP expression, DNA methylation, and tumor-infiltrating lymphocytes. (*A*) Heat map showing the correlation between SFRP1 transcript expression and tumor-infiltrating lymphocytes; (*B*) Heat map depicting the correlation between DNA methylation of SFRP1 and tumor-infiltrating lymphocytes; (*C*) Heat map illustrating the correlation between SFRP2 transcript expression and tumor-infiltrating lymphocytes; (*D*) Heat map presenting the correlation between DNA methylation of SFRP2 and tumor-infiltrating lymphocytes; (*E*) Heat map showing the correlation between the methylation levels of SFRP1/2 and the expression of CD4 + T cells, CD8 + T cells, macrophages, neutrophils, and B cells in colorectal cancer.

Using the TIMER database, we found that SFRP1 was positively correlated with CD4 and CD8 T cells, macrophages, and neutrophils, while SFRP2 showed similar positive correlations. Interestingly, data from the TISIDB database revealed that when SFRP1 was methylated, its methylation levels were strongly negatively correlated with CD4 and CD8 T cells, macrophages, neutrophils, and B cells. The results for SFRP2 mirrored those observed for SFRP1. These findings suggest that the DNA methylation of SFRP1 and SFRP2 may play a critical role in regulating immune cell infiltration in CRC (Fig. 10).

## Discussion

Currently, the role of the SFRP family in tumor pathogenesis remains poorly understood. Most studies have focused on the role of SFRPs as regulators of the WNT signaling pathway. SFRPs can influence tumor metastasis and invasion by inhibiting epithelial-mesenchymal transition, promoting cell apoptosis, and utilizing other mechanisms (Liang *et al*. 2019). Previous studies have reported differential expression of SFRPs in various types of tumors; however, their expression patterns in CRC have not been well characterized. In this study, we thoroughly analyzed the expression, prognosis, functional enrichment, immune cell infiltration, and methylation levels of the SFRP family in CRC. We also examined the relationship between SFRP1/2 expression and apoptosis and proliferation in CRC cells.

SFRP1, known as a tumor suppressor gene in most cancers, influences tumor development through epigenetic inactivation, which affects DNA methylation or miRNA-mediated transcriptional silencing. Additionally, SFRP1 plays a crucial role in regulating responses to chemotherapy and epigenetic modification drugs (Baharudin *et al*. 2020). Hypermethylation of the SFRP1 promoter is frequently observed in various cancers and is a common mechanism for downregulating this gene (Strzelczyk *et al*. 2019). For instance, it has been reported that SFRP1 methylation inhibits its expression and contributes to the pathogenesis of bladder cancer through the Wnt signaling pathway (Rogler *et al*. 2015).

In our study, we observed a significant decrease in SFRP1 expression in CRC tissue, likely due to methylation. We speculate that high-frequency methylation of SFRP1 may occur in CRC tissues, leading to reduced expression. Interestingly, we found no significant difference in OS or disease progression-free survival between patients with low and high SFRP1 expression. However, some studies have demonstrated that, in nasopharyngeal carcinoma, patients with low expression of SFRP1 exhibited significantly worse OS, DFS rate, and distant metastasis-free survival compared to patients with high expression of SFRP1 (Rogler *et al*. 2015). Davaadorj *et al*. reported that in hepatocellular carcinoma, the deletion of the SFRP1 gene was associated with a poor prognosis (Davaadorj *et al*. 2016). Patients with low SFRP1 expression showed larger tumor sizes and vascular invasion (Cheng et al. 2017a). Similarly, in glioblastoma multiforme, patients with low expression of SFRP1 had worse OS compared to those with positive SFRP1 expression, which is associated with a good prognosis (Majchrzak-Celińska *et al*. 2021). SFRP1 methylation has also been linked to ovarian cancer recurrence and shorter OS (Zhang *et al*. 2019). The lack of a significant association in the TCGA database could be due to an insufficient sample size or the absence of a relationship between SFRP1 methylation and prognosis. Therefore, future studies with larger sample sizes are needed to evaluate SFRP1 methylation in patients.

SFRP2 is frequently hypermethylated in many tumors, and its downregulation is closely associated with Wnt signaling activity and tumor progression (Wu *et al.* 2021). Most studies consider SFRP2 a tumor suppressor gene, although a few studies have suggested that SFRP2 may have a cancer-promoting effect on certain cancer types (Liu *et al*. 2017). Additionally, SFRP2 methylation has been shown to accelerate cancer cell invasion and tumor progression (Luo *et al*. 2016). SFRP2 can inhibit WNT-induced β-catenin dissociation, affecting cell cycle processes and tumor cell proliferation. Therefore, SFRP2 is considered an antagonist of the Wnt signaling pathway and a novel tumor suppressor. In gastric cancer, SFRP2 expression is significantly lower in tumor tissues compared to adjacent non-cancerous tissues. Stimulating SFRP2 expression in gastric cancer can inhibit tumor cell proliferation and induce apoptosis. Thus, SFRP2 methylation also plays a crucial role in gastric cancer development, and its hypermethylation is closely associated with low expression (Yan *et al*. 2021).

Our study found that SFRP2 expression was significantly lower in CRC tissues compared to normal tissues. We analyzed the GEPIA database to assess the prognostic value of SFRP2 in CRC patients. Our findings showed that patients with high SFRP2 expression had poorer OS rates and disease progression-free periods compared to those with low expression. These results contradict our findings of low SFRP2 expression in early-stage CRC. One possible explanation is that SFRP2 expression is initially enriched in the early stages of CRC. However, as the tumor microenvironment changes, extensive SFRP2 methylation occurs, leading to reduced tumor inhibition and promoting tumor development through various signaling pathways. Another possibility is that SFRP2 expression differs significantly due to various clinical interventions, which may affect the prognosis of certain patients. Therefore, while our research suggests that SFRP2 acts as a tumor suppressor gene in CRC, the specific mechanisms still require further clarification.

SFRP3 is primarily associated with bone development (Fang *et al*. 2021). Currently, there are limited studies on SFRP3, and its role remains largely unexplored. One study reported that SFRP3 may have a protective role in CRC patients in Saudi Arabia (Younis *et al*. 2022). However, we found no statistical difference in SFRP3 expression between CRC tissues and normal tissues. Furthermore, analysis of the GEPIA database indicated that SFRP3 expression does not affect OS or PFS in patients. SFRP4, another Wnt antagonist, is involved in cell proliferation, differentiation, and tumorigenesis. It was shown that SFRP4 can predict the survival time of patients with gastric cancer and ovarian cancer and is an important immune-related factor (Yu *et al*. 2022; Varier *et al*. 2023). In this study, we found that SFRP4 expression was highly expressed in CRC. However, SFRP4 expression did not have prognostic significance for determining the prognosis of CRC patients. Research on SFRP5 has primarily focused on cardiovascular and cerebrovascular diseases, with limited studies on its role in tumors (Tong *et al*. 2019). Some studies have also suggested that SFRP5, as a physiological tumor suppressor, has potential diagnostic and prognostic value in CRC, although the specific mechanisms have not been clarified (Kirana *et al*. 2020). In our study, we found that SFRP5 expression in CRC was lower than that in normal tissues, but there was no significant correlation between SFRP5 expression and DFS and OS in CRC.

The interaction network and enrichment analysis of the SFRP family molecules indicate that SFRPs and their interacting molecules are closely associated with the Wnt signaling pathway, playing significant roles in regulating the immune system and stem cell differentiation. Previous studies have shown that SFRP4 is associated with regulatory T cell (Treg) infiltration in pancreatic ductal adenocarcinoma (Yang *et al*. 2019). Recent preclinical and clinical evidence suggests that tumor-infiltrating immune cells may serve as effective targets for the diagnosis and treatment of CRC. However, the relationship between the SFRPs family and immune infiltration has yet to be extensively studied.

In our study, we investigated the correlation between SFRP1-5 and the tumor immune infiltration levels of five major immune cell types: B cells, CD8 + T cells, CD4 + T cells, macrophages, and neutrophils. The results showed a positive correlation between SFRP expression and the infiltration of CD4 + T cells and macrophages. These findings suggest that SFRPs may regulate the immune status of CRC patients and play an important role in the tumor immune microenvironment.

Previous studies have found a strong relationship between DNA methylation and immune invasion. For instance, Li *et al*. found that DNA methylation of NEFM in breast cancer is closely associated with immune invasion, which can affect the prognosis of breast cancer patients (Li *et al*. 2021a). Research on DNA methylation in lung adenocarcinoma has shown that it affects tumor immune cell infiltration and serves as a prognostic and predictive biomarker for patient outcomes and immunotherapy responses. Building on these findings, our study investigated the impact of SFRP1/2 on the prognosis of CRC patients and examined the correlation between SFRP1/2 expression and immune cell infiltration.

Through the TCGA database, we found a correlation between SFRP1/2 expression and their methylation levels, further confirming the relationship between SFRP1/2 and methyltransferases. Specifically, we observed a relationship between SFRP1/2 expression and the expression of the methyltransferases DNMT1, DNMT3A, and DNMT3B.

We predicted the presence of four CpG islands in SFRP1 and two CpG islands in SFRP2 using the MethPrimer online website to further study the methylation level of SFRP1/2. Through BSP and MSP experiments, we confirmed that the methylation level of SFRP1/2 in CRC was significantly increased compared to normal colorectal tissues and cells. To validate the relationship between SFRP1/2 methylation and immune cell infiltration, we used the TISIDB website and found a positive correlation between SFRP1/2 and the expression of tumor-infiltrating lymphocytes in CRC. Our results also demonstrate that the demethylation of SFRP1/2 can significantly suppress tumor cell proliferation and promote apoptosis. Therefore, further exploration of the specific mechanisms and functions of SFRP1/2 transcriptional expression and DNA methylation in regulating the tumor microenvironment is warranted.

However, this article mainly analyzes the role of the SFRP family in CRC, but there are still some limitations. For instance, the analysis of SFRP in CRC immune infiltration did not assess the degree of immune cell infiltration. Additionally, the subsequent basic experiments were limited to simple assessments of methylation and the effects of SFRP1/2 on CRC cell proliferation and apoptosis. Further research is needed to address these gaps and provide a more comprehensive understanding of SFRP’s role in CRC.

## Conclusion

The expression, methylation, and immune cell infiltration of the SFRP family in CRC were almost different compared to normal individuals. The expression levels of SFRP1, SFRP2, and SFRP5 were significantly reduced in CRC patients compared to normal individuals, while SFRP4 expression was elevated. Higher SFRP2 mRNA expression was strongly associated with better OS, disease-specific survival, and longer progression-free intervals. Furthermore, SFRP1 and SFRP2 expressions were associated with immune invasion, where higher levels were linked to increased immune infiltration. Both SFRP1 and SFRP2 showed hypermethylation in CRC. Silencing SFRP1/2 expression resulted in enhanced proliferation of CRC cells. The findings suggest that SFRPs could be potential therapeutic targets and key genes related to immune cell infiltration in CRC.

## Data Availability

All data are included in the manuscript. Upon requests to the corresponding author, additional data would be available. And the databases can be accessed through https://www.ncbi.nlm.nih.gov/gds.
